# Supplementary material for: A cointegration analysis of rabies cases and weather components in Davao City, Philippines from 2006 to 2017
Source: PLoS One. 2020 Aug 25;15(8):e0236278. doi: 10.1371/journal.pone.0236278 (PMC7446973; doi:10.1371/journal.pone.0236278)
Supplement: S1 Table — (DOCX) [file pone.0236278.s001.docx]

**S1 Table. Optimal lag-length selection for the positive rabies cases.**

|  | DF GLS tau Test Statistic | | | Critical Values | | |
| --- | --- | --- | --- | --- | --- | --- |
| [lags] | Rabies  Cases | Precipitation | Temperature | 1% | 5% | 10% |
| 13 | -1.014 | -2.646 | -2.442 | -3.527 | -2.780 | -2.505 |
| 12 | -1.330 | -2.62 | -2.245 | -3.527 | -2.800 | -2.524 |
| 11 | -1.702 | -2.788 | -2.039 | -3.527 | -2.819 | -2.542 |
| 10 | -1.677 | -2.572 | -2.109 | -3.527 | -2.838 | -2.560 |
| 9 | -1.839 | -3.058 | -2.384 | -3.527 | -2.857 | -2.577 |
| 8 | -2.181 | -3.449 | -2.959 | -3.527 | -2.875 | -2.594 |
| 7 | -2.044 | -3.505 | -3.658 | -3.527 | -2.892 | -2.610 |
| 6 | -2.216 | -3.659 | -3.584 | -3.527 | -2.909 | -2.625 |
| 5 | -2.818 | -4.132 | -3.375 | -3.527 | -2.924 | -2.639 |
| 4 | -3.477 | -4.193 | -3.435 | -3.527 | -2.939 | -2.652 |
| 3 | -4.037 | -4.467 | -4.02 | -3.527 | -2.952 | -2.665 |
| 2 | -4.722 | -5.344 | -6.047 | -3.527 | -2.965 | -2.676 |
| 1 | -7.007 | -7.235 | -6.344 | -3.527 | -2.977 | -2.687 |

Maxlag = 13 chosen by Schwarz criterion.
