# Supplementary material for: A cointegration analysis of rabies cases and weather components in Davao City, Philippines from 2006 to 2017
Source: PLoS One. 2020 Aug 25;15(8):e0236278. doi: 10.1371/journal.pone.0236278 (PMC7446973; doi:10.1371/journal.pone.0236278)
Supplement: S2 Table — (DOCX) [file pone.0236278.s002.docx]

**S2 Table. Lag-length selection for Johansen’s test for cointegration.**

| lag | LL | LR | df | p | FPE | AIC | HQIC | SBIC |
| --- | --- | --- | --- | --- | --- | --- | --- | --- |
| 1 | -702.4930 | . | 9 | . | 5.2100* | 10.1642* | 10.2410* | 10.3533* |
| 2 | -699.9680 | 5.0510* | 9 | 0.8300 | 5.7157* | 10.2567* | 10.4104* | 10.6349* |
| 3 | -693.2730 | 13.3890* | 9 | 0.1460 | 5.9093* | 10.2896* | 10.5202* | 10.8569* |
| 4 | -673.7310 | 39.0850* | 9 | 0.0000 | 5.0868* | 10.1390* | 10.4464* | 10.8954* |
